# Peripheral-central network analysis of cancer cachexia status accompanied by the polarization of hypothalamic microglia with low expression of inhibitory immune checkpoint receptors

**DOI:** 10.1186/s13041-024-01091-9

**Published:** 2024-04-29

**Authors:** Yukari Suda, Keiko Nakamura, Fukiko Matsuyama, Yusuke Hamada, Hitoshi Makabe, Michiko Narita, Yasuyuki Nagumo, Tomohisa Mori, Naoko Kuzumaki, Minoru Narita

**Affiliations:** 1 Department of Pharmacology, Hoshi University School of Pharmacy and Pharmaceutical Sciences, 2-4-41 Ebara, Shinagawa-Ku, Tokyo, 142-8501 Japan; 2 Division of Cancer Pathophysiology, National Cancer Center Research Institute, 5-1-1 Tsukiji, Chuo-Ku, Tokyo, 104-0045 Japan; 3 Department of Pharmacy, National Cancer Center Hospital, 5-1-1 Tsukiji, Chuo-Ku, Tokyo, 104-0045 Japan; 4 Japan Small Animal Medical Center, 1-10-4 Higashitokorozawawada, Tokorozawa-Shi, Saitama, 359-0023 Japan

**Keywords:** Cachexia, Inhibitory immune checkpoint receptors, Microglia, Hypothalamus, LPS, Lipocalin-2, Gut microbiota

## Abstract

While the excessive inflammation in cancer cachexia is well-known to be induced by the overproduction of inflammatory mediators in the periphery, microflora disruption and brain dysfunction are also considered to contribute to the induction of cancer cachexia. Hypothalamic microglia play a crucial role in brain inflammation and central-peripheral immune circuits via the production of inflammatory mediators. In the present study, we evaluated possible changes in excessive secretion of gut microbiota-derived endotoxin and the expression timeline of several inflammation-regulatory mediators and their inhibiting modulators in hypothalamic microglia of a mouse model of cancer cachexia following transplantation of pancreatic cancer cells. We demonstrated that the plasma level of lipopolysaccharide (LPS) was significantly increased with an increase in anaerobic bacteria, especially *Firmicutes*, in the gut at the late stage of tumor-bearing mice that exhibited dramatic appetite loss, sarcopenia and severe peripheral immune suppression. At the early stage, in which tumor-bearing mice had not yet displayed “cachexia symptoms”, the mRNA expression of pro-inflammatory cytokines, but not of the neurodegenerative and severe inflammatory modulator lipocalin-2 (LCN2), was significantly increased, whereas at the late “cachexia stage”, the level of LCN2 mRNA was significantly increased along with significant decreases in levels of inhibitory immune checkpoint receptors programmed death receptor-1 (PD-1) and CD112R in hypothalamic microglia. In addition, a high density of activated neurons in the paraventricular nucleus (PVN) of the hypothalamus region and a significant increase in corticosterone secretion were found in cachexia model mice. Related to the cachexia state, released corticosterone was clearly increased in normal mice with specific activation of PVN neurons. A marked decrease in the natural killer cell population was also observed in the spleen of mice with robust activation of PVN neurons as well as mice with cancer cachexia. On the other hand, in vivo administration of LPS in normal mice induced hypothalamic microglia with low expression of inhibitory immune checkpoint receptors. These findings suggest that the induction of cancer cachexia may parallel exacerbation of the hypothalamic inflammatory status with polarization to microglia expressed with low levels of inhibitory immune checkpoint receptors following LPS release from the gut microflora.

**Supplementary Information:**

The online version contains supplementary material available at 10.1186/s13041-024-01091-9.

## Introduction

Cancer cachexia is one of the most common complex metabolic abnormality syndromes in late-stage cancer patients [1, 2]. The European Palliative Care Research Collaborative (EPCRC) guidelines suggest that cachexia can be divided into three stages: pre-cachexia, cachexia, and refractory cachexia, and the life expectancy after progression to refractory cachexia is less than approximately 3 months [3]. Cachexia, which is typically associated with weight loss, anorexia, and sarcopenia-like symptoms, accounts for 20% of cancer deaths and is known to cause acute disability, including increased depression and anxiety [4, 5]. In addition, cachexia has been shown to affect postoperative outcomes, immune disorders, and the effectiveness of antineoplastic therapy [6–8]. Therefore, therapeutic intervention for cancer cachexia is necessary. However, therapeutic agents have not yet been established. In 2021, anamorelin, a novel ghrelin receptor agonist for cancer cachexia, was approved, but it is still insufficient to fundamentally improve the condition of patients with cancer cachexia [9]. Cancer cachexia is the most common symptom of pancreatic cancer and occurs in approximately 90% of patients [8]. In the present study, we attempted to produce a mouse model of cancer cachexia with mental and physical vulnerability by peritoneal dissemination of the mouse pancreatic cancer cell line Pan02.

Although the mechanisms underlying the induction of cachexia are not yet known, some central nervus system (CNS)-mediated cancer symptoms, such as anorexia and fatigue, support the idea that one of these mechanisms could be brain dysregulation during malignancy. Since cachexia has been shown to affect the expression of key metabolism-related factors in the brain, resulting in a reduction of the intracellular uptake of glucose, and promote an Alzheimer's disease-like neuroinflammatory pathway via circulating endotoxins, brain inflammation has been proposed to be one of its key drivers [10, 11]. Among brain regions, the hypothalamus mainly corresponds to cachexia, given its anatomic location and its ability to access peripheral signals directly due to attenuation of the blood–brain barrier. It has been widely accepted that microglia are a key player in mediating neuroinflammation related to brain dysfunction as well as the control of energy and appetite/feeding [12]. However, symptom/timeline-dependent changes in an aggravating inflammatory status and their mechanisms in the brain are not fully understood. On the other hand, the gut microbiota is known to profoundly influence many aspects of host physiology, including immune system development. Furthermore, previous studies on the gut-brain axis have shown that the gut microbiota could regulate brain function and behavior through the immune system [13]. Therefore, in this study, we examined whether the induction of cancer cachexia could be accompanied by an aggravating inflammatory status in hypothalamic microglia via the disruption of gut microflora-related malignancy.

## Materials and methods

### Animals

We used male C57BL/6 J mice (7–8 weeks old) (Tokyo Laboratory Animals Science Co., Ltd., Tokyo, Japan), cFos-tdTomato mice (16–18 weeks old) and CRH-ires-Cre mice [B6(Cg)-*Crh*^tm1(cre)Zjh^/J; Stock #012704, Jackson Laboratory, Bar Harbor, ME, USA] (16 weeks old) for this study. Male cFos-tdTomato mice were breeding cFos-2AiCreER^T2^ mice [C57BL/6-*Fos* ^*tm1(icreERT2)Phsh*^; Cyagen Biosciences Inc., Santa Clara, CA, USA, [14]] with LSL-tdTomato mice [B6.Cg-*Gt(ROSA)26Sor*^*tm14(CAG−tdTomato)Hze*^/J; Stock #007914, Jackson Laboratory]. Mice had access to food and water ad libitum in a temperature- and humidity-controlled room (24 ± 1 °C, 55 ± 5%, relative humidity) with a 12-h light–dark cycle (light on at 8 a.m.). All procedures for the animal study were performed during the light cycle at our laboratory at Hoshi University, following their Guiding Principles. All experimental procedures minimized the number and suffering of the animals. In this study, important adverse events were not observed in any of the experimental groups.

### Pan02 cell culture

Pan02-ffLuc cells that were genetically engineered to express fluorescent protein-fused luciferase (ffLuc) in mouse-derived pancreatic cancer cells (PAN 02, NCI DCTD Tumor Repository 0507794) were used for the experiment. The lentiviral vector CSII-EF-ffLuc containing the *ffLuc* gene (a Venus fluorescent protein [15] and firefly luciferase fusion gene) required for transformation was gifted by Drs. Hideyuki Okano (Keio University, Tokyo, Japan) and Hiroyuki Miyoshi (Keio University) [16]. Pan02-ffLuc cells were cultured in RPMI-medium (Sigma-Aldrich Co., St. Louis, MO, USA) supplemented with 10% fetal bovine serum (FBS; Thermo Fisher Scientific, Inc., Waltham, MA, USA), 1% penicillin–streptomycin (P/S; Thermo Fisher Scientific, Inc.) and 1% L-glutamine (Thermo Fisher Scientific, Inc.).

### Preparation of mouse models of cancer cachexia and evaluation of their behavioral phenotypes

Pan02-ffLuc cells were used to generate mouse models of cancer cachexia. Cells were harvested by treatment with trypsin, counted, and adjusted to 1.0 × 10^6^ cells/mL with Dulbecco's Phosphate Buffered Saline (PBS; Thermo Fisher Scientific, Inc.). One mL of the cell suspension was injected intraperitoneally into the abdominal cavity of mice. For the control group, 1 mL of PBS was injected intraperitoneally into the abdominal cavity of mice. We measured body weight and food intake at 1, 2 and 3 weeks after Pan02-ffLuc cell transplantation to evaluate the progression of cancer cachexia-like symptoms. In addition, physical function and muscle weakness, which are symptoms of sarcopenia, were assessed by grip strength tests and rotarod tests.

### Grip strength test

Using a grip strength meter (GPM-100B; MELQUEST, Toyama), we performed the grip strength test at 1, 2 and 3 weeks after Pan02-ffLuc cell transplantation. Measurements were taken three times and the average of the three values was calculated.

### Rotarod assay

The rotarod assay was performed according to a previously reported protocol [17]. Motor coordination was assessed using a rotarod (KN-75; Natsume Seisakusho Co., Ltd.). After mice were habituated to the apparatus, they were trained to ride the apparatus continuously at 4 rpm for 5 min. After pre-training, mice were individually placed on a slowly rotating bar (4 rpm/min) which was then continuously accelerated at 20 rpm/min; the time when the mice fell off the rod was recorded. The speed started at 4 rpm and increased by 1 rpm every 8 s until it reached 20 rpm. We set a cut-off of 300 s and ran the test three times.

### Bioluminescence imaging

Three weeks after Pan02-ffLuc cell transplantation, bioluminescence imaging was performed with an IVIS Lumina series III (Caliper Life Sciences, Almeda, CA). For noninvasive imaging, mice were administered intraperitoneally with d-luciferin at 4.5 mg/mouse (15 mg/mL, FUJIFILM Wako). Seven min after receiving d-luciferin, mice were anesthetized in a chamber with 3% isoflurane and imaged. For ex *vivo* imaging of individual organs, organs were taken out from mice 7 min post administration of d-luciferin, and immediately imaged.

### Bioplex analysis

Plasma samples were taken from cancer cachexia mice at 24 h after the evaluation of behavioral phenotypes. Cytokines, chemokines, and growth factors in plasma samples were quantified by the Bio-Plex® Multiplex System (Bio-Rad Laboratories Inc., CA, USA). In this study, the Bio-Plex Pro™ Mouse Cytokine 23-plex Assay (#64,140,214, Bio-Rad Laboratories Inc.) and Bio-Plex Pro™ Mouse Cytokine 9-plex Assay (#64,174,947, Bio-Rad Laboratories Inc.) were used.

### Drug treatment

Tamoxifen (Sigma) was dissolved at 20 mg/mL in ethanol and then corn oil (FUJIFILM Wako)] was added to give a final concentration of 10 mg/mL tamoxifen. The ethanol was then evaporated by vacuum under centrifugation. Lipopolysaccharide (LPS, 600 μg/mouse) was administered intravenously and the hypothalamus was sampled 12 h later. Clozapine N-oxide (CNO; 3 mg/kg, Abcam plc., Cambridge, UK) to activate Gq*-*coupled human M3 muscarinic receptor (hM3Dq) was dissolved in saline. CRH-Cre mice injected AAV10-hSyn-Flex-hM3Dq-mCherry were sampled 30 min after CNO administration.

### Flow cytometry

Cytotoxic immune cells in the spleen were sorted according to a previously reported protocol [18]. Twenty-four hr after the evaluation of behavioral phenotypes, the spleen was isolated from mice at the pre-cachexia or cachexia stage, and then homogenized with PBS (Themo Fisher Scientific Inc.). To remove cell aggregates, the homogenized suspension was passed through a 100 μm cell strainer. Subsequently, ammonium chloride was applied to a single-cell suspension for hemolysis, and the cell suspension was fractionated at 6 × 10^6^ cells/tube. After blocking, cells were stained with the following antibodies: T cells (CD45.2-APC/Cy7, CD4-FITC, CD8-PE) and natural killer (NK) cells (CD45.2-APC-Cy7, CD3ε-PE, CD49b-APC). All antibodies were purchased from Bio Legend (San Diego, CA, USA). Immune cells were sorted using a BD FACS Aria™ II Cell Sorter (BD Biosciences) and then analyzed. For mice with treatment of LPS or saline, flow cytometry procedure was carried out 12 h after each treatment.

### Endotoxin quantitation

Endotoxin (LPS) levels in plasma samples were measured using the Pierce™ Chromogenic Endotoxin Quant Kit (Thermo Fisher Scientific). Briefly, plasma samples, standards and blanks were incubated at 37℃ with the amebocyte lysate reagent and chromogenic substrate. After adding of 25% acetic acid to stop the reaction, the absorbance was determined at 405 nm.

### Fecal sampling and bacterial flora analysis

At 3 weeks after Pan02 cell transplantation, fecal samples were collected under aseptic conditions for accurate analysis of the bacterial flora. The feces were spontaneously excreted in trays in plastic cages, collected using sterile tweezers and promptly frozen. DNA was extracted from the feces and analyzed for bacterial flora (Repertoire Genesis, Osaka, Japan). The V3-V4 regions of the 16S ribosomal RNA (rRNA) gene in the extracted DNA were amplified by polymerase chain reaction (PCR) and the amplified PCR products were sequenced using a MiSeq (Illumina, San Diego, CA, USA). The sequence data obtained were pre-processed and subjected to phylogenetic and flora identification analyses using Flora Genesis software (Repertoire Genesis Co., Osaka).

### Sorting of CD11b^+^ microglial cells

Sorting of CD11b^**+**^ microglial cells was performed according to a previously reported protocol [17]. Mice at the pre-cachexia or cachexia stage were sacrificed at 24 h after the evaluation of behavioral phenotypes. Mice were perfused with PBS containing 5 IU/ml heparin, and brains were dissected and processed into a single-cell suspension according to the manufacturer’s protocols. To obtain CD11b-positive microglial cells, tissue samples from the hypothalamus region of mice were processed to give single cells using an adult brain dissociation kit (Miltenyi Biotec., Bergisch Gladbach, Germany). Subsequent experiments were conducted using samples taken from 3 mice as one sample. Biotin micro beads (Miltenyi Biotec Co.) conjugated to anti-astrocyte cell surface antigen-2 (ACSA2) were added to the cells, and the ACSA2^**+**^ cell fraction was removed as ACSA2^**+**^ astrocyte cells using an auto magnetic-activated cell sorting (MACS) pro separator. Then, CD11b (microglia) micro beads (Miltenyi Biotec Co.) were added to the ACSA2-negative cell fraction and the CD11b^**+**^ cell fraction was sorted by an auto MACS pro separator.

### Reverse transcription-quantitative PCR (RT-qPCR)

RT-qRCR was performed according to a previously reported protocol [17, 18]. Total RNAs were isolated from immune cells and CD11b^+^ microglia of the mice using a mirVana miRNA Isolation Kit (Thermo Fisher Scientific Inc.), and then first-strand cDNAs were synthesized. qPCR was performed using a StepOne Plus™ System (Thermo Fisher Scientific Inc.) and synthesized primers (Supplementary Table 1). Glyceraldehybe-3-phosphate dehydrogenase (Gapdh) was used as a normalization control.

### TRAP labeling and histology

Eighteen days after the Pan02 cell transplantation, cFos-tdTomato mice were subcutaneously injected with 100 mg/kg tamoxifen to capture activated neurons under the cachexia condition. cFos-tdTomato mice at 3 weeks after Pan02 cell transplantation were transcardially perfusion-fixed with 4% paraformaldehyde in PBS under anesthesia with isoflurane (3%, inhalation). Post-fixed coronal brain sections including paraventricular nucleus (PVN) were cryoprotected in 20–30(w/v)% sucrose and embedded in optimal cutting temperature compound (O.C.T. compound; Tissue Tek; Sakura Fine Technical). The brain sections were cut on a cryostat (CM1860; Leica Microsystems, Heidelberg, Germany) (10 µm). The tdTomato fluorescence was detected using a light microscope (BX-53; Olympus) and photographed with a digital camera (MD-695; Molecular Devices). The images were analyzed using MetaMorph software (Molecular Devices).

### Stereotaxic adeno-associated virus (AAV) injection

Robust activation of corticotropin releasing hormone (CRH)^PVN^ neuron by a designer receptor exclusively activated by designer drugs (DREADD) system was performed according to a previously reported protocol [19]. For manipulation of CRH neurons in the PVN, AAV10-hSyn-Flex- hM3Dq-mCherry was bilaterally injected into the PVN of male CRH-ires-Cre mice (A/P, − 0.69 mm; M/L, ± 1.06 mm from the bregma; D/V, − 4.66 mm from the dura; with a 10° angle toward the midline in the coronal plane) through an internal cannula (Eicom Co., Kyoto, Japan) at a rate of 0.25 μL/min for 4 min (1 μL total volume per side) and an air pressure injector system (Micro-syringe Pump-Model ESP-32; Eicom Co.). The virus used in this study was a kind gift from Dr. Akihiro Yamanaka (Nagoya University, Nagoya, Japan). Cre-dependent expression of mCherry was histologically verified through microscopic analysis of brain sections containing the PVN.

### Corticosterone enzyme-linked immunosorbent assay (ELISA)

Blood plasma corticosterone levels were measured using the Corticosterone ELISA kit (Enzo Life Science) according to a previously reported protocol [19]. The blood sample was centrifuged at 5000 rpm for 5 min at room temperature, and the upper layer was then gently collected.

### Statistical analysis

We analyzed and described the statistical significance of differences between groups according to an unpaired *t*-test, two-way and one-way analysis of variance (ANOVA) with the Bonferroni multiple comparisons test. The data were subjected to a comparative analysis by testing the null hypothesis for the Pearson product moment correlation. All statistical analyses were performed by Prism version 8.0 (GraphPad software, La Jolla, CA, USA).

## Result

### Generation of a mouse model of peritoneal dissemination in pancreatic cancer using Pan02 cells

**Fig. 1:**
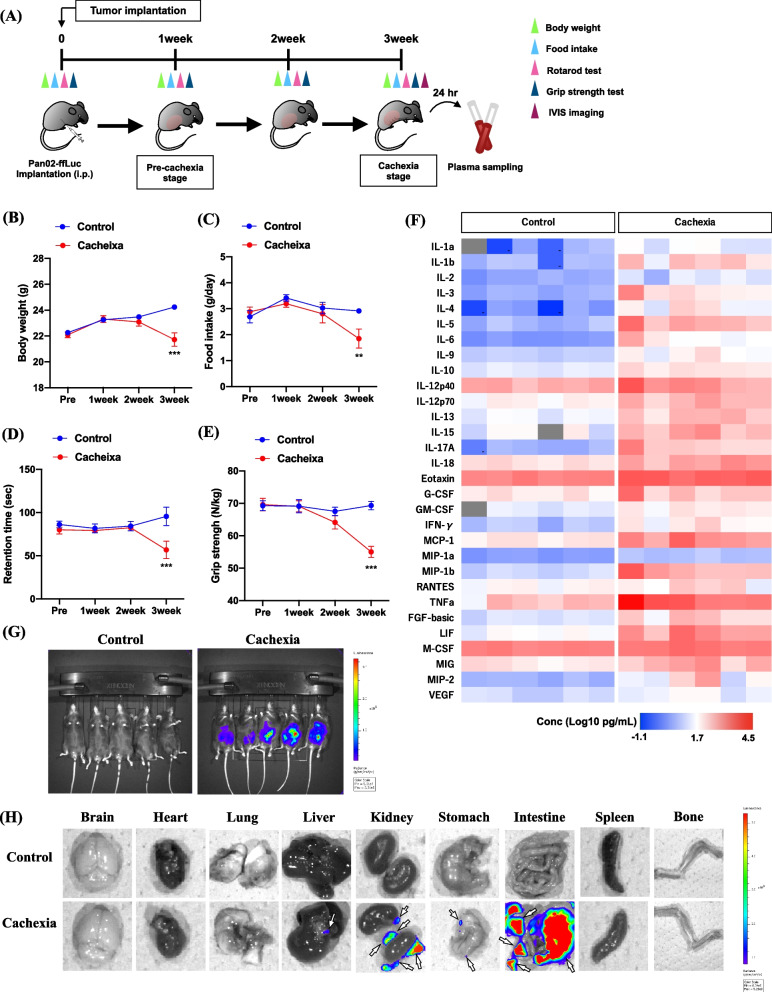
Generation of a mouse model of peritoneal dissemination of pancreatic cancer using Pan02-ffLuc cells. **A** Schedule and schematic workflow of behavioral experiments to assess cachexia-like symptoms after implantation of Pan02-ffLuc cells. **B**-**E** Time-course changes in body weight (**B**), food intake (**C**), rotarod performance (**D**) and grip strength (**E**) of cancer cachexia-model mice after implantation of pancreatic cancer cells compared to those of control mice. Each value represents the mean ± S.E.M. Statistical analysis was performed using two-way ANOVA with the Bonferroni test: **p < 0.01, ***p < 0.001 vs. control group (*n* = 9–11). **F** A heatmap of the inflammatory cytokine level [log _10_ (concentration (pg/mL)] at the cachexia stage after implantation of Pan02-ffLuc cells evaluated by multiplex cytokine assay (*n* = 6). **G** The detection of intra-peritoneal tumor engraftment and growth by IVIS imaging of the luciferase signal in mice at the cachexia stage after implantation of pancreatic cancer cells (*n* = 5). **H** Ex vivo IVIS imaging of major organs in control and cancer cachexia model mice. White arrows represent the detection of luciferase signal as cancer cell growth

First, we generated a mouse model of cancer cachexia by the intraperitoneal injection of cells from the murine pancreatic cancer line Pan02 (Fig. 1A). The timeline of the experiment to assess cancer cachexia-like symptoms is shown in Fig. 1A. While there was no change in body weight or food intake for 1 or 2 weeks, significant decreases in weight gain and food intake were noted at 3 weeks after pancreatic cancer cell implantation compared to those in control mice (Fig. 1B, C, two-way ANOVA with the post-hoc Bonferroni test, **p < 0.01, ***p < 0.001 vs. control group). Furthermore, sarcopenia symptoms, as indicated by a shortened retention time in the rotarod test and decreased muscle strength by the grip strength test, were found to occur at 3 weeks, but not at 1 and 2 weeks following the implantation of pancreatic cancer cells (Fig. 1D, E, two-way ANOVA with the post-hoc Bonferroni test, ***p < 0.001 vs. control group). We also observed a robust increase in plasma levels of various cytokines and chemokines in a mouse model with peritoneal dissemination of pancreatic cancer cells at the cachexia stage, when cachexia-like symptoms were observed (Fig. 1F). Furthermore, we confirmed using the IVIS imaging system that the transplanted pancreatic cancer cells spread throughout the subperitoneal tissue in mice at the cachexia stage (Fig. 1G). The transplanted pancreatic cancer cells were also found to have metastasized to the liver, kidney, stomach and intestine, but not to the brain, heart, lung, spleen or bone (Fig. 1H).

### Changes in gut microbiota and blood LPS levels under cancer cachexia

**Fig. 2:**
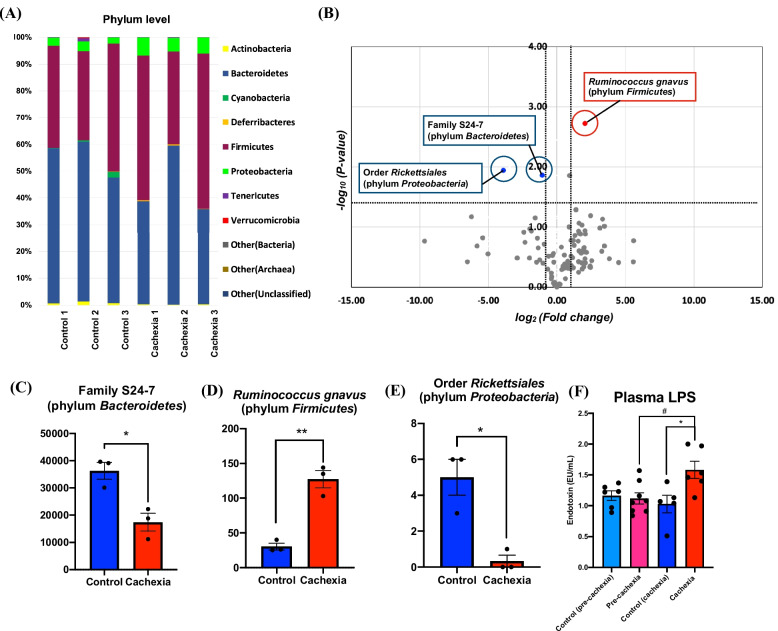
Changes in gut microbiota and blood LPS levels under the condition of cancer cachexia. **A** Gut microbiota composition at the phylum level in control and cancer cachexia-model mice (*n* = 3). **B** Volcano plots showing differential microbiota that are upregulated (red) or downregulated (blue) in cancer cachexia-model mice with a significant *p* value < 0.05 and fold change < 0.5 or > 2 when compared with control mice (*n* = 3). **C**-**E** The number of leads of family S24-7 (phylum *Bacteroidetes*) (**C**), *Ruminococcus gnavus* (phylum *Firmicutes*) (**D**) and Order *Rickettsiales* (phylum *Proteobacteria*) (**E**) in cancer cachexia-model mice compared to control mice. Each column represents the mean ± S.E.M. Unpaired* t*-test: *p < 0.05, **p < 0.01 vs. control group (*n* = 3). **F** Plasma endotoxin (LPS) levels in mice at the pre-cachexia or cachexia stage after transplantation of pancreatic cancer cells. Each value represents the mean ± S.E.M. The data were subjected to a comparative analysis by one-way ANOVA with the Bonferroni test: ^#^*p* < 0.05 vs. pre-cachexia group, *p < 0.05 vs. control (cachexia) group (*n* = 5–8)

Recently, several associations have been reported between various diseases and gut microbiota [13]. To clarify the relationship of gut microbiota to cancer cachexia, we analyzed 16S rRNA microbiota using feces collected from cancer cachexia model mice at 3 weeks after pancreatic cancer cell transplantation. The results showed that the feces were mostly composed of bacteria belonging to *Bacteroidetes* and *Firmicutes*, and no significant differences in the intestinal microbiota at the Phylum level were observed between the control and cancer cachexia groups (Fig. 2A). Therefore, we created volcano plots of the variation in the expression of individual bacterial species in feces and identified the species that varied between the control and cancer cachexia groups (Fig. 2B). We found a significant decrease in family S24-7 (phylum *Bacteroidetes*), which is important for intestinal barrier function [20], and a significant increase in *Ruminococcus gnavus* (phylum *Firmicutes*), which is the main component of intestinal mucus, and causes the mucosal layer to weaken [21], in the cancer cachexia model mice compared to the control group (Fig. 2C, D, Unpaired *t*-test, *p < 0.05, **p < 0.01 vs. control group). Although the number of leads was relatively low, a significant decrease in Order *Rickettsiales* (phylum *Proteobacteria*), the function of which has not yet been investigated, was also observed in the cancer cachexia group (Fig. 2E, Unpaired *t*-test, *p < 0.05 vs. control group). Under these conditions, we observed a significant increase in LPS levels in the plasma of a mouse model at the cachexia stage, but not pre-cachexia stage (Fig. 2F, one-way ANOVA with the post-hoc Bonferroni test, ^#^*p* < 0.05 vs. pre-cachexia group, *p < 0.05 vs. control (cachexia) group).

### Changes in hypothalamic microglia in a mouse model of peritoneal dissemination in pancreatic cancer

**Fig. 3:**
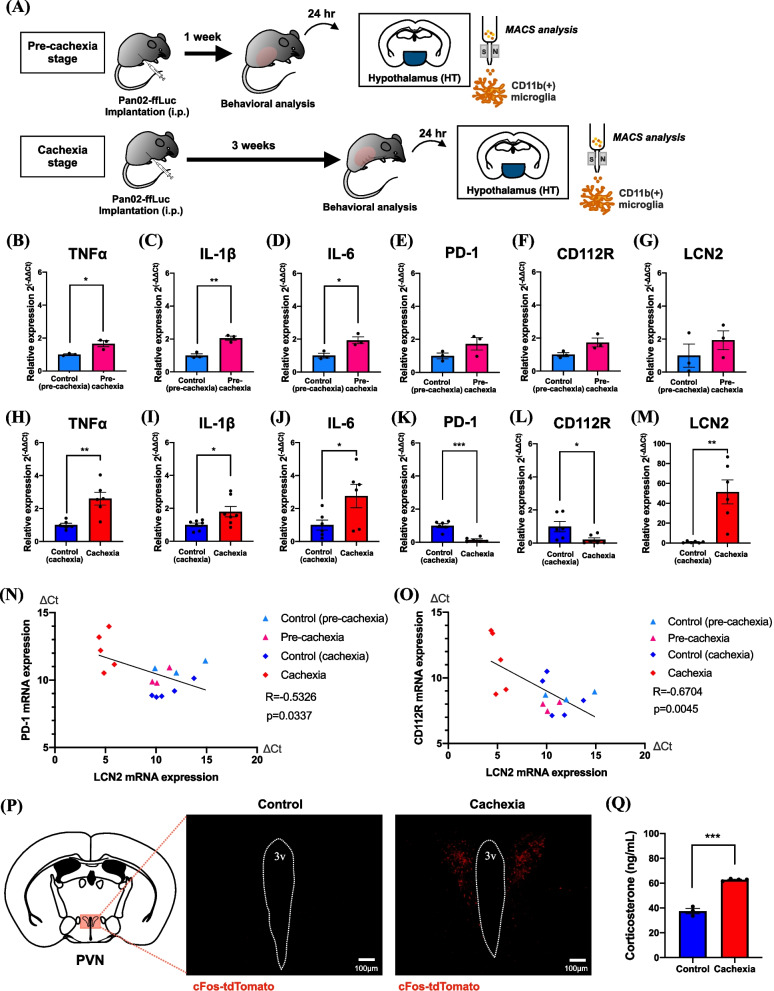
Changes in hypothalamic microglia in a mouse model of peritoneal dissemination of pancreatic cancer. **A** Schematic workflow for the isolation of hypothalamic microglia of pancreatic cancer cell-transplanted mice at the pre-cachexia or cachexia stage. **B**-**G** Changes in the mRNA expression of TNFα, IL-1β, IL-6, PD-1, CD112R and LCN2 in the hypothalamic CD11b^+^ microglia of pancreatic cancer cell-transplanted mice at the pre-cachexia compared to those of control mice. Data are plotted as mean ± S.E.M. Unpaired *t*-test: *p < 0.05, **p < 0.01 vs. control group (3 independent experiments). **H**-**M** Changes in the mRNA expression of TNFα, IL-1β, IL-6, PD-1, CD112R and LCN2 in the hypothalamic CD11b^+^ microglia of pancreatic cancer cell-transplanted mice at the cachexia stage. Data are plotted as mean ± S.E.M. Unpaired *t*-test: *p < 0.05, **p < 0.01, ***p < 0.001 vs. control group (6 or 7 independent experiments). **N** Pearson correlation scatter plot of the expression of LCN2 mRNA (ΔCt: LCN2_(Ct)_-GAPDH_(Ct)_) and the expression of PD-1 mRNA (ΔCt: PD-1_(Ct)_-GAPDH_(Ct)_) in hypothalamic microglia obtained from pancreatic cancer cell-transplanted mice at the pre-cachexia or cachexia stage. (*r* = -05326, *p* = 0.0337) (**O**) Pearson correlation scatter plot of the expression of LCN2 mRNA (ΔCt: LCN2_(Ct)_-GAPDH_(Ct)_) and the expression of CD112R mRNA (ΔCt: CD112R_(Ct)_-GAPDH_(Ct)_) in hypothalamic microglia obtained from pancreatic cancer cell-transplanted mice at the pre-cachexia or cachexia stage. (*r* = -0.6704, *p* = 0.0045) (**P**) Increase in the number of cFos-tdTomato^+^ cells in the PVN of pancreatic cancer cell-transplanted cfos-TRAP2-tdTomato mice at the cachexia stage (on Day 18) compared to that in control mice (Scale bar: 100 μm). **Q** Plasma levels of corticosterone in mice at the cachexia stage after transplantation of pancreatic cancer cells. Each value represents the mean ± S.E.M. Unpaired* t*-test: ***p < 0.001 vs. control group (*n* = 3–4)

To investigate the characteristics of microglia in the brain, we isolated CD11b-positive microglia from the hypothalamus of a mouse model of peritoneal dissemination using auto MACS (Fig. 3A). We confirmed using fluorescence activated cell sorting (FACS) analysis that little of CD45^high^ myeloid cells was penetrated after systemic perfusion (data not shown). The results of temporal changes in inflammatory cytokines in hypothalamic CD11b-positive microglia of control and pancreatic cancer cell-transplanted mice showed a high-level expression of tumor necrosis factor-α (TNFα), interleukin (IL)-1β and IL-6 at the pre-cachexia and cachexia stage (Fig. 3B-D, H-J, Unpaired *t*-test, *p < 0.05, **p < 0.01 vs. control group). On the other hand, an analysis of changes in immune checkpoint receptors over time showed significant decreases in the expression of immune checkpoint receptors programmed death receptor-1 (PD-1) and CD112R in hypothalamic microglia at the cachexia stage, but not at the pre-cachexia stage (Fig. 3E, F, K-L, Unpaired *t*-test, *p < 0.05, ***p < 0.001 vs. control group). Furthermore, a significant increase in the expression of the inflammation-inducing secreted protein lipocalin 2 (LCN2) was similarly observed in hypothalamic microglia at the cachexia stage, but not at the pre-cachexia stage (Fig. 3G, M, Unpaired *t*-test, **p < 0.01 vs. control group). A negative correlation was observed between PD-1 or CD112R mRNA expression and LCN2 mRNA expression in hypothalamic microglia at the pre-cachexia and cachexia stage after transplantation of pancreatic cancer cells (Fig. 3N, R=-0.5326, *p* = 0.0337, Fig. 3O; *R* = -0.6704, *p* = 0.0045). LCN2 has been reported to activate hypothalamic PVN neurons via melanocortin 4 receptor (MC4R) [22]. Therefore, we investigated whether cancer cachexia model mice could exhibit the activation of hypothalamic neurons by using the TRAP method. As a result, we found a high-density distribution of c-fos-positive activated neurons in the PVN of cFos-TRAP2-tdTomato mice at 3 weeks after transplantation of pancreatic cancer cells (Fig. 3P). Furthermore, in such cancer cachexia model mice, we detected a high plasma concentration of corticosterone, which can be released by activating the hypothalamic–pituitary–adrenal (HPA) axis to control systemic immunity (Fig. 3Q, Unpaired *t*-test, ***p < 0.001 vs. control group).

### Changes in cytotoxic immune cells of the spleen in a mouse model of peritoneal dissemination in pancreatic cancer

**Fig. 4:**
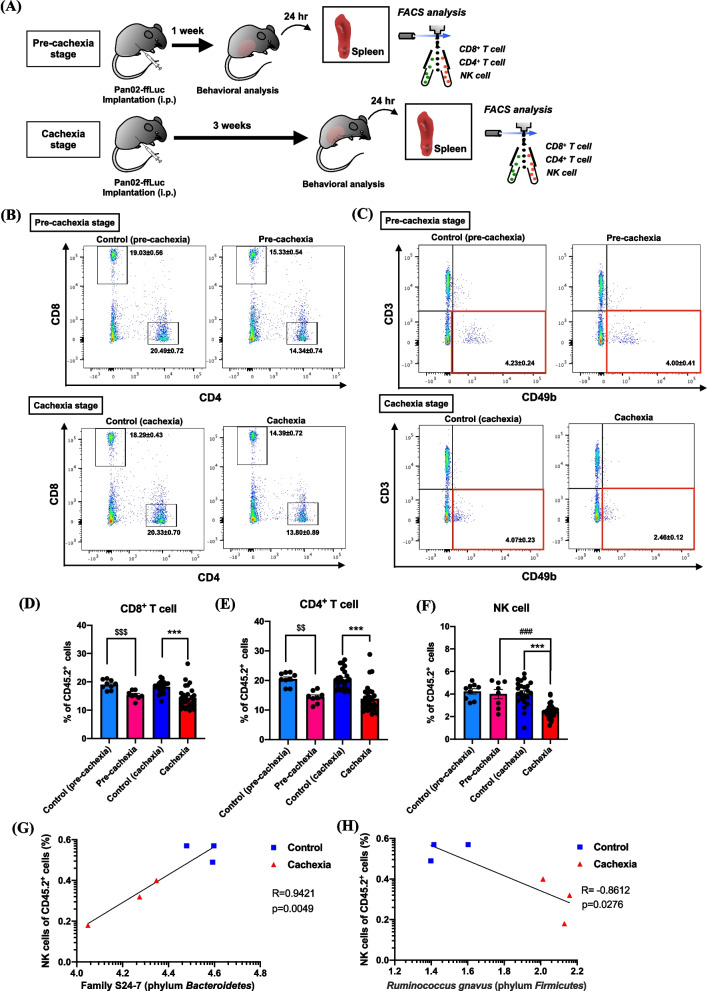
Changes in cytotoxic immune cells of the spleen in a mouse model of peritoneal dissemination of pancreatic cancer. **A** Schematic workflow for the isolation of cytotoxic immune cells (CD8^+^ T cells, CD4^+^ T cells and NK cells) of pancreatic cancer cell-transplanted mice at the pre-cachexia or cachexia stage. **B**, **C** Representative flow cytometric plots of CD8^+^ T cells, CD4^+^ T cells (**B**) and NK cells (**C**) of cancer cachexia-model mice at the pre-cachexia or cachexia stage compared to that of control mice. **D**-**F** Time-course changes in the populations of CD8^+^ T cells (**D**), CD4^+^ T cells (**E**) and NK cells (**F**) of mice after implantation of pancreatic cancer cells compared to those of each control mice. Each value represents the mean ± S.E.M. The data were subjected to a comparative analysis by one-way ANOVA with the Bonferroni test: ^$$^*p* < 0.01, ^$$$^*p* < 0.001 vs. control (pre-cachexia) group, ^###^*p* < 0.001 vs. pre-cachexia group, ***p < 0.001 vs. control (cachexia) group (*n* = 8–28). **G** Pearson correlation scatter plot of the expression of family S24-7 (phylum *Bacteroidetes*) and the population of NK cells in the spleen obtained from pancreatic cancer cell-transplanted mice at the cachexia stage. (*r* = 0.9421, *p* = 0.0049) (**H**) Pearson correlation scatter plot of the expression of *Ruminococcus gnavus* (phylum *Firmicutes*) and the population of NK cells in the spleen obtained from pancreatic cancer cell-transplanted mice at the cachexia stage. (*r* = -0.8612, *p* = 0.0276)

Since hypothalamic microglial inflammation is assumed to promote the HPA axis and affect the immune system, we next investigated changes in cytotoxic immune cells of the spleen in a mouse model of peritoneal dissemination of pancreatic cancer cells at the pre-cachexia or cachexia stage (Fig. 4A). The results showed that the percentages of CD8^+^ T and CD4^+^ T cells to CD45.2^+^ cells were significantly decreased at the the pre-cachexia and cachexia stage after transplantation of pancreatic cancer cells (Fig. 4B, D, E, one-way ANOVA with the post-hoc Bonferroni test, ^$$^*p* < 0.01, ^$$$^*p* < 0.001 vs. control (pre-cachexia) group, ***p < 0.001 vs. control (cachexia) group). On the other hand, the percentage of NK cells was significantly reduced at the cachexia stage, but not at the pre-cachexia stage, after transplantation of pancreatic cancer cells (Fig. 4C, F, one-way ANOVA with the post-hoc Bonferroni test, ^###^*p* < 0.001 vs. pre-cachexia group, ***p < 0.001 vs. control (cachexia) group). A positive linear correlation was significantly observed between the percentage of NK cells in the spleen and the expression of family S24-7 (phylum *Bacteroidetes*) at the cachexia stage (Fig. 4G; *R* = 0.9421, *p* = 0.0049). Moreover, a negative linear correlation was significantly observed between the percentage of NK cells in the spleen and the expression of *Ruminococcus gnavus* (phylum *Firmicutes*) at the cachexia stage after transplantation of pancreatic cancer cells (Fig. 4H; *R* = -0.8612, *p* = 0.0276).

### Decrease in NK cells through the activation of CRH^PVN^ neurons

**Fig. 5:**
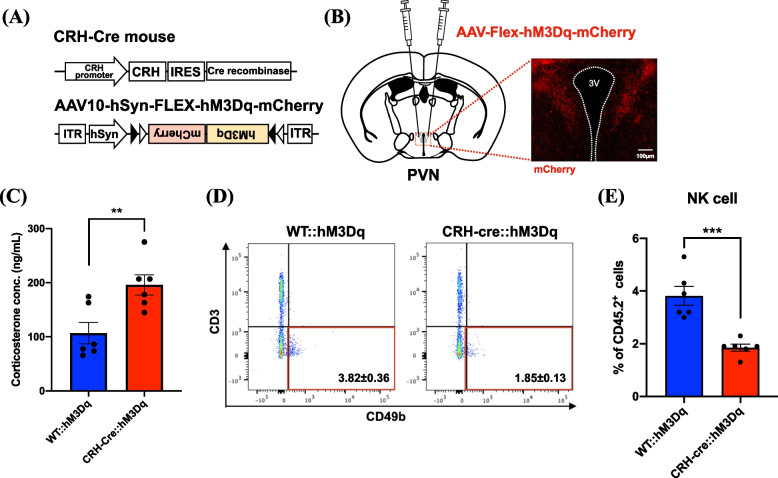
Changes in NK cells of the spleen by the specific activation of CRH^PVN^ neurons. **A** Schematic illustration of gene constructs of CRH-Cre mice and AAV-Flex-hM3Dq-mCherry vector. **B** Representative image showing mCherry-expressing neurons in the PVN of CRH-Cre mice with the PVN microinjection of AAV-Flex-hM3Dq-mCherry (Scale bar: 100 μm). **C** The plasma level of corticosterone in the CRH-Cre::hM3Dq mice at 30 min after CNO-induced activation of CRH^PVN^ neurons. Data are plotted as mean ± S.E.M. Unpaired* t*-test: **p < 0.01 vs. WT::hM3Dq group (*n* = 6). **D**, **E** Representative flow cytometric plots (**D**) and quantitative evaluation of the number (**E**) of NK cells in the spleen of CRH-Cre::hM3Dq mice at 30 min after CNO-induced activation of CRH^PVN^ neurons compared to those in WT mice. Data are plotted as mean ± S.E.M. Unpaired* t*-test: ***p < 0.001 vs. WT::hM3Dq group (*n* = 6)

We next evaluated whether activation of CRH^PVN^ neurons could change cytotoxic immune cells in the spleen using the DREADD system. To positively control the activity of CRH^PVN^ neurons, the microinjection of AAV vector Cre-dependently expressing hM3Dq was performed into the PVN of CRH-Cre mice (Fig. 5A, B). By robust activation of CRH^PVN^ neurons, the plasma concentration of corticosterone in CRH-Cre::hM3Dq mice was significantly increased compared to that in WT::hM3Dq mice (Fig. 5C, Unpaired *t*-test, **p < 0.01 vs. WT::hM3Dq group). Under these conditions, similar to the results in cancer cachexia-model mice, the percentages of NK cells were significantly decreased in the spleen of CRH-Cre::hM3Dq mice (Fig. 5D, E, Unpaired *t*-test, *** *p* < 0.001 vs. WT::hM3Dq group).

### Changes in cytotoxic immune cells in the spleen and hypothalamic microglia induced by LPS administration

**Fig. 6:**
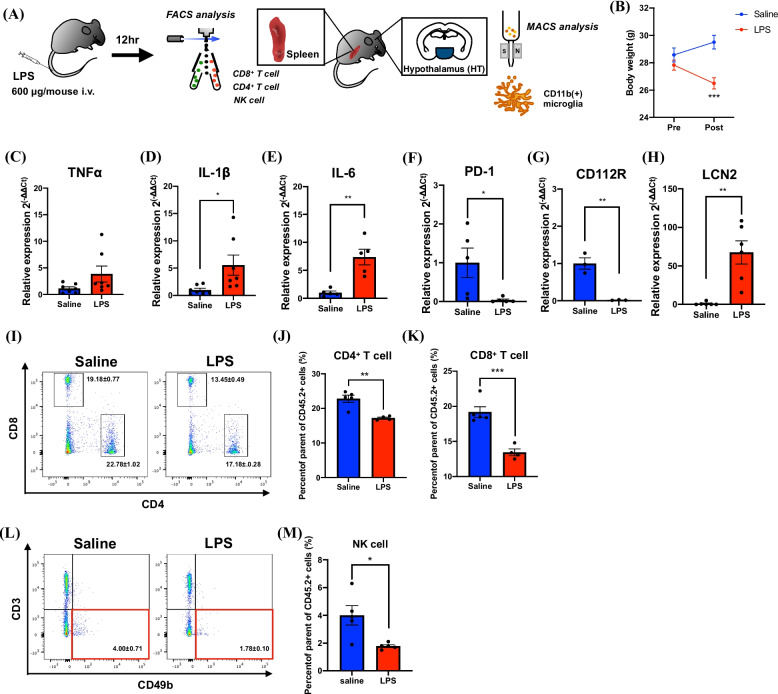
Changes in cytotoxic immune cells of the spleen and hypothalamic microglia of mice with LPS-injected systemic inflammation. **A** Schedule of FACS and MACS sorting after LPS injection (600 μg/0.1 mL/mouse, i.v.). **B** Changes in body weight at pre/post-injection of saline or LPS. Data are plotted as mean ± S.E.M. Statistical analysis was performed using two-way ANOVA with the Bonferroni test: ***p < 0.001 vs. saline-injected group (*n* = 5–6). **C**-**H** Changes in the mRNA expression of TNFα, IL-1β, IL-6, PD-1, CD112R and LCN2 in the hypothalamic CD11b^+^ microglia of LPS-injected model mice compared to those in saline-injected mice. Data are plotted as mean ± S.E.M. Unpaired *t*-test: *p < 0.05, **p < 0.01 vs. saline-injected group (3 or 7 independent experiments). (I-M) Representative flow cytometric plots (left panel) and quantitative evaluation of the number (right panel) of CD8^+^ T cells, CD4^+^ T cells (I-K) and NK cells (L, M) in the spleen of LPS-injected model mice compared to those in saline-injected mice. Data are plotted as mean ± S.E.M. Unpaired *t*-test: *p < 0.05, **p < 0.01, ***p < 0.001 vs. saline-injected group (*n* = 4–5)

To elucidate the effect of LPS on the transformation of cytotoxic immune cells or hypothalamic microglia, we injected LPS intravenously into normal mice (Fig. 6A). Weight loss was observed in LPS-injected mice (Fig. 6B, two-way ANOVA with the post-hoc Bonferroni test, *** *p* < 0.001 vs. saline-injected group). The TNFα, IL-1β, and IL-6 were significantly increased in hypothalamic CD11b^+^ microglia of LPS-injected mice similar to the cancer cachexia-model mice (Fig. 6C-E, Unpaired *t*-test, * *p* < 0.05, **p < 0.01vs. saline-injected group). Furthermore, the mRNA levels of PD-1 and CD112R were significantly reduced in CD11b-positive microglia in the hypothalamus of LPS-injected mice (Fig. 6F, G, Unpaired *t*-test, *p < 0.05, **p < 0.01 vs. saline-injected group). Under these conditions, LCN2 was significantly increased in CD11b-positive microglia in the hypothalamus of LPS-injected mice (Fig. 6H, Unpaired *t*-test, ** *p* < 0.01 vs. saline-injected group). Furthermore, similar to the cancer cachexia-model mice, the percentages of CD8^+^ T cells, CD4^+^ T cells (Fig. 6I-K) and NK cells (Fig. 6L-M) were significantly decreased in the spleen of LPS-injected mice (Unpaired *t*-test, *p < 0.05, **p < 0.01, *** *p* < 0.001 vs. saline-injected group).

## Discussion

Cachexia is thought to reflect the onset of unsustainable metabolic demands on the body because of persistent disease. A growing amount of evidence suggests that this deleterious response results from the disruption of brain function [23]. In this study, we created a mouse model of cachexia by peritoneal dissemination of pancreatic cancer cells (Pan02 cells) and investigated the timeline changes in the expression of several inflammatory mediators and immune checkpoint molecules in hypothalamic microglia 1 week (pre-cachexia stage) and 3 weeks (cachexia stage) later. No major changes in food consumption, body weight, or sarcopenia-like symptoms were detected at 1 week after transplantation of pancreatic cancer cells. Comparatively, there were marked decreases in weight gain and food intake, accompanied by motor dysfunction and muscle weakness 3 weeks after transplantation of pancreatic cancer cells. Furthermore, a cytokine storm in the plasma was clearly detected at the cachexia stage. Under the present condition, the transplanted pancreatic cancer cells spread throughout the subperitoneal tissue without metastasis to the brain and spleen.

To clarify the possible relationship between cancer cachexia and intestinal microbiota, we first analyzed 16S rRNA microbiota using feces collected from cancer cachexia model mice. According to a 16S rRNA analysis which found that the feces were mostly composed of bacteria belonging to *Bacteroidetes* and *Firmicutes*, no significant differences in the gut microbiota at the portal level were observed between the control and cancer cachexia groups. However, family S24-7 (phylum *Bacteroidetes*), which is important for intestinal barrier function [20], was significantly reduced and *Ruminococcus gnavus* (phylum *Firmicutes*), which is the main component of intestinal mucus and responsible for mucosal fragility [21], were significantly increased at the cachexia stage. Furthermore, the plasma level of endotoxin (LPS), a major component of the outer membrane of Gram-negative bacteria, was significantly increased at the cachexia stage, but not at the pre-cachexia stage. It has been reported that released LPS and inflammation markers are positively correlated with the level of *Ruminococcus gnavus* (phylum *Firmicutes*) [24]. Taken together, these results suggest that the disruption of the intestinal barrier function associated with changes in the gut microbiota may leak LPS into the plasma at the cachexia stage.

Using these mice, we next isolated CD11b-positive microglia from the hypothalamus to analyze the timeline changes in the expression of inflammatory cytokines. The results showed a sustained increase in levels of the mRNA expression of TNFα, IL-1β, and IL-6 in hypothalamic microglia, which started from the pre-cachexia stage and lasted for the cachexia stage after pancreatic cancer cell transplantation. These results support the idea that a cytokine storm could be induced in the hypothalamus before cachexia-like symptoms are present. We also analyzed the possible changes in the expression of immune checkpoint receptors in hypothalamic microglia of mice with pancreatic cancer cells spreading to the peritoneum. We found that mRNA levels of PD-1 and CD112R did not change significantly at the pre-cachexia stage after the transplantation of pancreatic cancer cells spreading to the peritoneum, whereas significant and dramatic decreases in these levels were observed at the cachexia stage. We should emphasize here that the mRNA expression of LCN2, a proinflammatory secretory protein [25], was robustly increased at the cachexia stage, but not the pre-cachexia stage, after transplantation of pancreatic cancer cells. Our findings are consistent with the previous finding that cachectic mice showed an increase in LCN2 expression in hypothalamic microglia and endothelial cells [26]. Interestingly, a negative correlation between PD-1 mRNA expression and LCN2 mRNA expression in hypothalamic microglia was likely to be observed under the progression of cachexia. In T cells, PD-1 reduces the activation of transcription factors such as activator protein 1 (AP1), nuclear factor of activated T cells (NFAT) and NF-κB via the tyrosine dephosphatase Src homology region 2 domain-containing phosphatase 2 (SHP-2) [27]. PD-1 has been shown to influence osteogenic/tooth differentiation of stem cells from the epiphyseal papilla by activating SHP2 signals and inducing the inhibition of NF-κB signals [28]. It has been recognized that stimulation of CD112R as well as PD-1 in T cells or NK cells clearly suppresses cytotoxic ability of immune cells via the activation of SHP2-NF-κB pathways [29, 30]. On the other hand, the expression of LCN2 is known to be markedly increased by NF-κB [31–33]. In this study, a marked decrease in PD-1 and CD112R mRNA expression was observed in hypothalamic microglia of cachexia models. Although a more comprehensive analysis will be required, the present findings suggest that the reduction of inhibitory immune checkpoint receptors may induce NF-κB activation, leading to an increase in LCN2 expression. It has been shown that the expression of LCN2 is increased under conditions of neuroinflammation in the brain, such as Alzheimer's disease [25, 34]. LCN2 is also known to act as an appetite suppressor and to produce multiple chemokines [35]. In fact, Olson et al. have demonstrated that intracerebroventricular administration of LCN2 induces weight loss and decreased food intake [35]. Furthermore, experiments using LCN2 knockout mice have shown that suppression of LCN2 can alleviate the cachexia phenotype of decreased food intake. In addition, LCN2 has been reported to activate hypothalamic PVN neurons via MC4R [22]. In the present study, we found a high density of activated neurons in the PVN region of cachexia model mice. Activation of PVN neurons by LCN2 is thought to alter immunity by overexciting the HPA axis with increasing corticosterone secretion in mice. Interestingly, we observed that released corticosterone was clearly increased in both mice with cachexia and normal mice with robust activation of the HPA axis. Related to this phenomenon, a significant and marked decrease in the NK cell population was observed in the spleen, a secondary lymphoid organ, of either mice that had displayed cachexia or normal mice with robust activation of the HPA axis. Taken together, these findings support the idea that induction of cachexia-like symptoms can be, at least partly, accompanied by the exacerbation of immunosuppression due to the hyperactivation of the HPA axis originating from PVN neurons.

Finally, we demonstrated that intravenous LPS administration significantly decreased the populations of CD8-positive T cells, CD4-positive T cells and NK cells in the spleen. Furthermore, a decreased expression of PD-1 and CD112R and an increased expression of LCN2 were detected in hypothalamic microglia after administration of LPS to normal mice. Taken together, these findings suggest that peritoneal disseminated pancreatic cancer may induce a disruption of the gut microbiota, resulting in increased LPS in the blood and the hypothalamus. This phenomenon may in turn activate inflammatory microglia in the hypothalamus with decreasing the expression of inhibitory immune checkpoint receptors, leading to the attenuation of immunity due to the overexcitation of the HPA axis.

As mentioned above, we found a marked increase in LCN2 expression along with a shut-down of PD-1/CD112R expression only at the most advanced stage of cachexia, during which we observed anorexia and sustained increases in cytokine production that began in the pre-cachexia stage. Therefore, we hypothesize that sustained cytokine production may overproduce LCN2 with a reduction of inhibitory immune checkpoint receptors in hypothalamic microglia, and then lead to the anorexia and sarcopenia along with the exacerbation of immunosuppression through the overexcitation of the HPA axis.

Tumors are known to compromise the integrity of the blood–brain barrier (BBB), and although limited, some myeloid cells have been shown to infiltrate the brain of a mouse model of pancreatic ductal adenocarcinoma [36]. Therefore, even though peripheral myeloid cells were not detected under the present condition, we cannot completely deny the possibility that the present CD11b-positive cells include infiltrating peripheral immune cells, such as monocyte-derived macrophages [37]. In general, the expression of inhibitory immune checkpoint receptors is thought to be elevated in peripheral immune cells when cancer becomes malignant [38]. Although further investigation is needed, we presume that the event of reduced expression of inhibitory immune checkpoint receptors observed in the present study may be almost caused in hypothalamic microglia.

In summary, these results suggest that cancer cachexia may be partly induced in association with deteriorating hypothalamic inflammation with the polarization to microglia accompanied by the low expression of inhibitory immune checkpoint receptors following LPS release from the gut microflora. It has been reported that a fiber-rich (FR) diet increases family S24-7 and contributes to the retention of the colonic mucus layer [20]. As well as a FR diet, probiotics, LPS inhibitors or blockade of the HPA axis may prevent the transition to the “cachexia stage” and help to treat the pathology of cancer cachexia.

### Supplementary Information

**Supplementary Material 1.**

## Data Availability

Data will be made available on reasonable request.

## Abbreviations

AP1: Activator protein 1
AAV: Adeno-associated virus
ANOVA: Analysis of variance
ACSA2: Astrocyte cell surface antigen-2
BBB: Blood–brain barrier
CD: Cluster of Differentiation
CNO: Clozapine N-oxide
CNS: Central nervus system
CRH: Corticotropin releasing hormone
DREADD: Designer receptor exclusively activated by designer drugs
ELISA: Enzyme-linked immunosorbent assay
EPCRC: European Palliative Care Research Collaborative
FACS: Fluorescence activated cell sorting
FBS: Fetal bovine serum
FR: Fiber-rich
Gapdh: Glyceraldehybe-3-phosphate dehydrogenase
hM3Dq: Gq*-*coupled human M3 muscarinic receptor
HPA: Hypothalamic–pituitary–adrenal
IL: Interleukin
ffLuc: Fluorescent protein-fused luciferase
LPS: Lipopolysaccharide
LCN2: Lipocalin-2
MACS: Magnetic-activated cell sorting
MC4R: Melanocortin 4 receptor
NFAT: Nuclear factor of activated T cells
NF-κB: Nuclear factor-kappa B
NK: Natural killer
O.C.T. compound: Optimal cutting temperature compound
PBS: Phosphate buffered saline
PCR: Polymerase chain reaction
PD-1: Programmed death receptor-1
PI: Propidium iodide
PVN: Paraventricular nucleus
rRNA: Ribosomal RNA
S.E.M.: Standard error of the mean
SHP-2: Src homology region 2 domain-containing phosphatase 2
TNFα: Tumor necrosis factor-α
