# Supplementary material for: Peripheral-central network analysis of cancer cachexia status accompanied by the polarization of hypothalamic microglia with low expression of inhibitory immune checkpoint receptors
Source: Mol Brain. 2024 Apr 29;17:20. doi: 10.1186/s13041-024-01091-9 (PMC11059753; doi:10.1186/s13041-024-01091-9)
Supplement: Supplementary file 1 — Supplementary Material 1. [file 13041_2024_1091_MOESM1_ESM.pdf]

**Table 1. Primer sequences used for RT-qPCR analysis**

| Symbol | Forward primer (5’→3’) | Reverse primer (5’→3’) |
|---|---|---|
| GAPDH | CATGGCCTTCCGTGTTCCTA | GATGCCTGCTTCACCACCTT |
| TNFα | GGCAGGTTCTGTCCCTTTCA | GGAGTGCCTCTTCTGCCAGTT |
| IL-1β | TCGTGCTGTCGGACCCATAT | TGTCGTTGCTTGGTTCTCCTT |
| IL-6 | CTGGTCTTCTGGAGTACCATAGC | TCTGTGACTCCAGCTTATCTCTTG |
| Pvrig (CD112R) | TCTCTGGCTATGACGGCAAC | TGGGAAAGTCACCCGTGTTC |
| PD-1 | GCAATCAGGGTGGCTTCTAGA | TTGGACAAGCTGCAGGTGAA |
| LCN2 | CCAGTTCGCCATGGTATTTT | CACACTCACCACCCATTCAG |
